# Experimental and mechanistic research on methane adsorption in anthracite modified by electrochemical treatment using selected electrode materials

**DOI:** 10.1038/s41598-019-53840-9

**Published:** 2019-11-20

**Authors:** Xiaoyu Zhang, Runxu Zhang, Tianhe Kang, Yaoqing Hu, Chao Li

**Affiliations:** Key Laboratory of In-situ Property-improving Mining of Ministry of Education, Taiyuan University of Technology, Taiyuan, 030024 P.R. China

**Keywords:** Mineralogy, Corrosion

## Abstract

The strong adsorption capacity of methane in anthracite can seriously affect the methane extraction. Electrochemical treatment is an effective way to weaken the capacity of methane adsorption in coal. Iron, copper, aluminum and graphite as four kinds of electrode materials were selected to modify anthracite by electrochemical treatment. The adsorption of methane in anthracite, before and after modification, was tested under different adsorption pressure. Based on the changes of pore characteristics and chemical groups of anthracite, the modification process of different electrode materials was analyzed. The results showed that after electrochemical modification, the adsorption of methane decreased, when the graphite electrode was used, the methane adsorption decreases the most, followed by copper and iron electrodes, and the aluminum electrode decreased the least. After electrochemical modification using aluminum, iron, copper and graphite electrodes, the Langmuir constant *a* reduced by 5.22%, 8.48%, 9.24% and 11.33%, respectively, and the degree of reduction is graphite > copper > iron > aluminum. After electrochemical modification using the graphite electrode, the Langmuir constant *b* was reduced by 23.52%. On the contrary, after electrochemical modification using the mental electrodes, the Langmuir constant *b* was increased by about 5%. The surface free energy of anthracite decreased with the adsorption of CH_4_, the higher the pressure, the more the free energy decreased, and the reduction of surface energy decreased after electrochemical modification. The difference of the electrode reactions was the main reason for the electrochemical results, the M^n+^ ions generated in the anode changed the properties of the clay mineral in the coal, and the H^+^ ions corroded the calcite minerals in the coal. The results obtained from this work indicate that the selection of electrode materials is crucial for the electrochemical modification, and graphite electrode is optimum for anthracite when accelerating methane extraction by electrochemical method.

## Introduction

The high degree of metamorphism and wildly development of micro-pore in anthracite lead to strong adsorption capacity and low desorption rate of methane, which seriously restrict the efficiency of methane extraction. Thus, researchers around the world have conducted numerous studies focused on the change of methane adsorption/desorption characteristic in coal, such as pressure relief of reservoirs^1^, and the application of heated hydro-fracture technology^2^, sound field effect^3^, electric field effect^4^, the action of electromagnetic field^5^ and electrochemical treatment^6^ optimizes methane adsorption/desorption performance in coal and helps to promote the methane extraction. The existing researches show that electrochemical treatment can significantly change the methane adsorption/desorption and seepage characteristics in coal^6–8^. Guo *et al*. based on electrochemical removal of carbonate and sulfide minerals, confirmed the effect of electrochemical treatment can unblock the mineral-filled fracture in the coal samples and increase pore volume and porosity. As a result, the average desorption rate increased by 68% during the period of reaching desorption equilibration after modification. However, only graphite electrode was mentioned in the electrochemical modification of anthracite, the influence of different electrode materials on the electrochemical modification effect was not considered.

There is relatively limited literature on reducing the adsorption characteristics of methane in coal using different electrodes when electrochemical method was used. However, studies focused on sulphur removal^9^, dewatering of fine coals^10^, electro-osmotic strengthening of silts^11^ have shown that electrode material is one of the key factors influencing electrochemical effects. During the electrochemical experiments, electrode material that commonly used includes non-inert metals (iron, copper, aluminum, etc.), inert metals (gold, silver, platinum, etc.), carbonaceous materials (graphite, etc.) and electrokinetic geosynthetics^12^ (EKGs). For the optimization of electrode material, scholars have carried out enormous comparative experimental studies. On one hand, some scholars suggested that the non-inert metals were better than that of carbon materials such as graphite. Segall *et al*. pointed out that the iron electrode performs better than the graphite electrode under the same energy consumption in the electroosmotic experiment^13^. Mohamedelhassan *et al*. also found that graphite electrodes had greater loss of energy and lower efficiency than metal electrodes^14^. Copper and stainless steel were considered the best choice for metal electrodes, and the advantages of copper electrodes lie in low cost, and the copper oxide formed by oxygen near copper electrodes can also conduct electricity and reduce the voltage loss at the anode. The stainless steel has good corrosion resistance^15^. On the other hand, some scholars suggested that traditional metal electrodes had many disadvantages such as electrode corrosion, exhaust difficulty, contact lost between electrodes and soil during electroosmosis^16^. Lefebvre and Jayasekera both reported that corrosion of steel and mild steel electrodes reduced the efficiency of electrochemical action^17,18^. Zhou *et al*. observed that graphite electrode appears to be a good option compared with iron electrode and copper electrode under high potential gradient (1.58 V/cm) with sludge from Hangzhou^11^, Suits also found that graphite was a more effective electrode material^19^.

Different experimental conditions caused different results of optimization of the electrode materials. Experimental samples obtained from all over the world differ greatly in mineral type, particle size and salt content, which generating different electrolyte environments in the electrochemical process. Lockhart compares the electroosmotic effects of different electrode materials in Cu clays and Al clays, and concluded that copper electrode behaves better than the graphite electrode in Cu clays, while in the Al clays, and the aluminum electrode performs better under the low potential gradient, and the graphite exhibits better performance under the high potential gradient^20^. As far as the electrode material is concerned, Mohamedelhassan pointed out that the potential loss of iron electrode was smaller than copper because the electrochemical potential of iron was lower than copper^14^ (electrochemical potential of iron, copper and graphite are −0.44 V, +0.34 V and +1.18 V respectively), the lower the electrochemical potential of the material, the stronger its reducibility, so the higher the activity, the easier the electrode reaction occurs.

In summary, the electrode materials have a profound influence on the effects of electrochemical modification, however, the optimization of the electrode materials still need further discussion. Therefore, this paper selected iron, copper, aluminum and graphite as four electrodes for electrochemical modification in anthracite, the adsorption of methane in coal samples were tested. To explain the mechanism of modification the pore structure, and chemical groups of the coal samples were also tested by Brunauer-Emmett-Teller (BET) gas adsorption technique, SEM-EDS analysis and FTIR measurement.

## Experiment

### Sample preparation

The test anthracite samples were prepared from the Sihe coal mine from the Qinshui coal field. The coal samples were sealed and stored underground and sent to the laboratory as soon as possible to avoid the physicochemical change caused by air oxidation. The mean maximum vitrinite reflectance (R_o,max_) and the maceral composition of the anthracite samples were tested following the standards of GB/T 6948-2008 and GB/T 8899-2013, respectively. The proximate analysis including moisture, ash, and volatile matter content was also conducted following the GB/T 212-2008 standard. The analysis results were listed in Table 1.

**Table 1:** Petrologic Characteristics and Proximate Analysis of Anthracite Sample.

R_o,max_ (%)	Proximate analysis (wt, %)			Ultimate analysis (%)			
	Moisture	Ash yield	Volatile matter	C	H	O	S
2.86	1.65	5.21	6.12	86.52	2.64	6.83	3.32

### Electrochemical modification of anthracite by different electrodes

**Figure 1:**
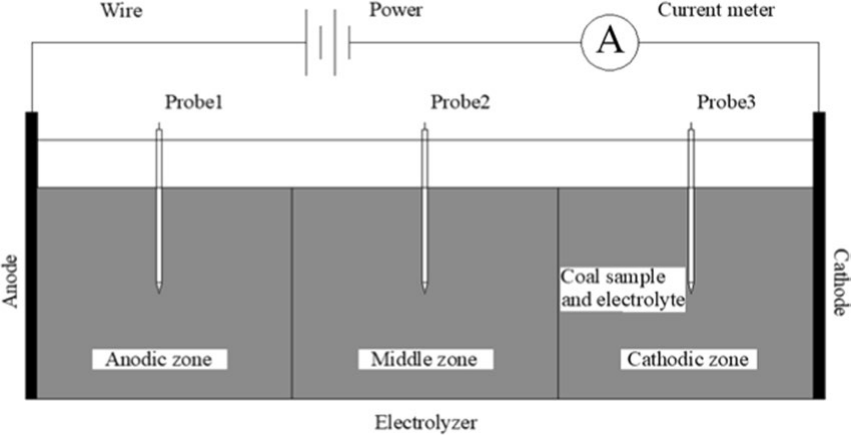
Schematic of the electrochemical modification device.

According to GB/19560-2008, before modification, the coal samples were processed into fine particles of 0.18–0.25 mm and dried in a vacuum oven at 373.15–378.15 K until a constant weight was achieved, then the coal samples were sufficiently saturated in Na_2_SO_4_ solution with a concentration of 0.05 mol/L, and the potential gradient was set to 4 V/cm, and the modification time was 120h^21^. The prepared coal samples were modified in the device as shown in Fig. 1. The device is mainly composed of an electrolyzer, a DC power supply, an ammeter, two electrode plates and potential probes. The electrolyzer is made of Plexiglas plates with 5 mm thick, the inner dimensions are 110 mm long, 60 mm wide, and 80 mm high. DC power was supplied by DH1722A-2 produced by Dahua Radio Instrument Factory, the output voltage range is 0–110 V, and the output current is 0–3 A.

### Methane desorption measurement

The methane adsorption in anthracite, before and after modification, were test by 3H-2000PHD manufactured by Beishide Instrument, the adsorption temperature was set to 313.15 K, and the maximum adsorption pressure was set to 4 MPa. Before testing, the coal samples were degassed in vacuum at 378.15 K, the degassing time was 300 minutes, and the constant time was 120 minutes after degassing. The equilibrium time of adsorption is 1800 s and the equilibrium standard deviation is 0.008 MPa.

### Micropore characterization

The micropore structures of the anthracite samples, before and after electrochemical modification, were analyzed low-pressure N_2_ adsorption by ASAP2020HD88 adsorption apparatus provided by Micromeritics. The surface area and pore volume were obtained by the Brunauer-Emmett-Teller (BET) model.

### SEM-EDS measurement

Scanning electron microscopy (SEM, Tescan Mira3, Czech), coupled with energy dispersive spectrometry (EDS, X-MaxN,UK), was used to achieve the morphology of the coal samples. Before testing, the coal samples were sputter coated with gold by E-1045 ion bean sputtering instrument provided by Hitachi.

### FTIR measurement

The Nicolet iS5 FTIR instrument (Thermo Fisher, USA) was employed to investigate the functional group distributions of the original and electrochemical modified anthracite samples. The collected spectral range of the instrument is 400–4000 cm^−1^ wavenumber. Before the test, the fine coal samples and Potassium bromide (KBr) were tableted in a ratio of 1:150 wt%.

## Results

### Methane adsorption

T1-T5 showed the experimental results of methane adsorption of coal samples before and after electrochemical modification using aluminum, iron, copper, and graphite electrodes. The results were listed in Table 2. Coal sample without modification has strong capacity of methane adsorption. When the pressure is 1, 2, 3 and 4 MPa, the amount of gas adsorption is 22.38 ml/g, 27.64 ml/g, 31.61 ml/g and 33.15 ml/g, respectively. After electrochemical modification using metal electrodes, the methane adsorption was decreased. The gas adsorption was further reduced after electrochemical modification using graphite electrode, when the pressure is 1, 2, 3 and 4 MPa, the adsorption of methane is 17.40 ml/g, 22.49 ml/g, 26.35 ml/g and 27.91 ml/g, respectively. The results indicated that after electrochemical modification, the methane adsorption in anthracite decreased, when the graphite electrode was used, the methane adsorption decreases the most, followed by copper and iron electrodes, and the aluminum electrode decreased the least.

**Table 2:** Experimental results of methane adsorption (ml/g).

Test number (Electrode material)	Sample mass (g)	Adsorption pressure (MPa)			
		1	2	3	4
T1(No electrode)	21.46	22.38	27.64	31.61	33.15
T2(Aluminum)	20.41	21.71	26.59	30.27	31.70
T3(Iron)	20.35	21.02	25.64	29.22	30.63
T4(Copper)	21.40	20.79	25.44	28.99	30.35
T5(Graphite)	20.61	17.40	22.49	26.35	27.91

**Figure 2:**
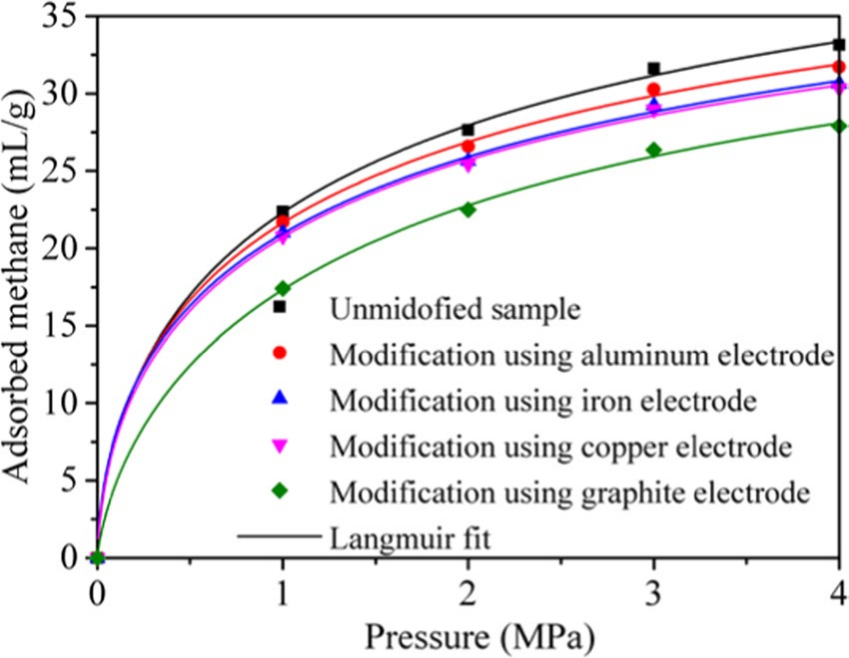
CH_4_ adsorption in anthracite after modification using different electrodes at a temperature of 313.15 K.

To investigate the rate of adsorption, the experimental results of methane adsorption were fitted by the Langmuir equation^22^:

1 $${\rm{V}}=\frac{{V}_{L}P}{({P}_{L}+P)}$$

where *V* is the amount of adsorbed gas, ml/g; *V*_*L*_ is the maximum amount of adsorbed gas, also known as Langmuir volume, which depends on the properties of adsorbent and adsorbate, indicating the limit adsorption capacity per unit mass solid at a given temperature, ml/g; *P*_*L*_ is adsorption constant, MPa^−1^; and *P* is the pressure, MPa. Figure 2 presented the fitting curves, *V*_*L*_ and *P*_*L*_ are called Langmuir constants, set *V*_*L*_ = *a*, 1/*P*_*L*_ = *b*, and the fitting Langmuir constants are listed in Table 3. From Fig. 2, we can determine that the gas adsorption of the coal samples, before and after modification, follows the Langmuir isotherm, and the amount of absorbed gas is unmodified raw coal > electrochemical modification using aluminum electrode > electrochemical modification using iron electrode > electrochemical modification using copper electrode > electrochemical modification using graphite electrode. The repeatability of the trend as shown in supplementary file.

**Table 3:** Langmuir constant extracted by fitting the Langmuir isotherm model.

Test number (Electrode material)	a(ml/g)	b(MPa^−1^)	Correlation coefficient R^2^
T1(No electrode)	39.920	1.220	0.998
T2(Aluminum)	37.836	1.284	0.998
T3(Iron)	36.536	1.286	0.998
T4(Copper)	36.232	1.283	0.998
T5(Graphite)	35.398	0.933	0.997

The maximum amount of methane adsorbed is determined by the natural characteristics of the coal, such as the coal rank and the porosity. Table 2 indicates that the maximum adsorption amount *a* of the unmodified coal is 39.920 ml/g, and the value of *a* is reduced after electrochemical modification using different electrodes. After electrochemical modification using aluminum, iron, copper and graphite electrodes, *a* reduced to 37.836 ml/g, 36.536 ml/g, 36.232 ml/g and 35.398 ml/g, respectively, decreased by 5.22%, 8.48%, 9.24% and 11.33%. We can determine that the properties of the coal samples changed after electrochemical modification, and the capacity of gas adsorption decreased. Adsorption constant *b* is determined by the potential well of methane adsorption of coal and the temperature of the adsorption system. The adsorption constant *b* of the unmodified coal was 1.220 MPa^−1^, after modified using the metal electrodes the value of *b* increased. When electrochemical modification using the aluminum, iron and copper electrodes, *b* raised to 1.284 MPa^−1^, 1.286 MPa^−1^ and 1.283 MPa^−1^ respectively, all increased by about 5%. However, *b* reduced to 0.933 MPa^−1^ after electrochemical modification using graphite electrode, decreased by 23.52% compared to the unmodified coal. During the test, the temperature of the adsorption system is constant, thus, the potential well increased when metal electrodes were used, and decreased using the graphite electrode.

### Surface free energy

The Langmuir formula was used to describe the adsorption process, and combined with Gibbs adsorption formula, and the change of surface energy of coal samples before and after electrochemical modification could be estimated. According to the theory of surface chemistry, when CH_4_ gas adsorbs on the coal surface, the concentration of CH_4_ gas in the coal surface area is higher than that in the coal structure, the difference is called surface excess Γ:

2 $$\Gamma =\frac{V}{{V}_{0}S}$$

where Γ is the surface excess, mol/m^2^; V is the gas adsorption capacity, L; V_0_ is the adsorption volume of gas, under the standard condition is 22.4 L/mol; S is the specific surface area of coal, m^2^/g. When coal adsorbs CH_4_ gas, the surface tension decreases. According to Gibbs formula:

3 $$-d{\rm{\gamma }}=\mathrm{RT}\Gamma d(\mathrm{ln}\,P)$$

where γ is surface tension, J/m^2^; R is universal gas constant, 8.3143 J/mol·K; T is absolute temperature, K; P is CH_4_ gas pressure, Pa. When introducing formula (2) into (3) and integrating pressure from 0 to P, we can get that:

4 $$\Delta {\rm{\gamma }}={{\rm{\gamma }}}_{0}-{\gamma }_{1}=\frac{RT}{{V}_{0}S}{\int }_{0}^{P}\,\frac{V}{P}dP$$

where γ_0_ is the surface tension of coal in vacuum, J/m^2^; and γ_1_ is the surface tension after CH_4_ adsorption, J/m^2^. The adsorption of CH_4_ in coal conforms to Langmuir adsorption Eq. (1), the fitting parameters a and b are obtained as shown in Table 3. By introducing formula (1) into (4), the following results are obtained:

5 $$\Delta {\rm{\gamma }}=\frac{RT}{{V}_{0}S}{\int }_{0}^{P}\,\frac{ab}{1+bP}dP=\frac{aRT}{{V}_{0}S}\,\mathrm{ln}(1+bP)$$

**Figure 3:**
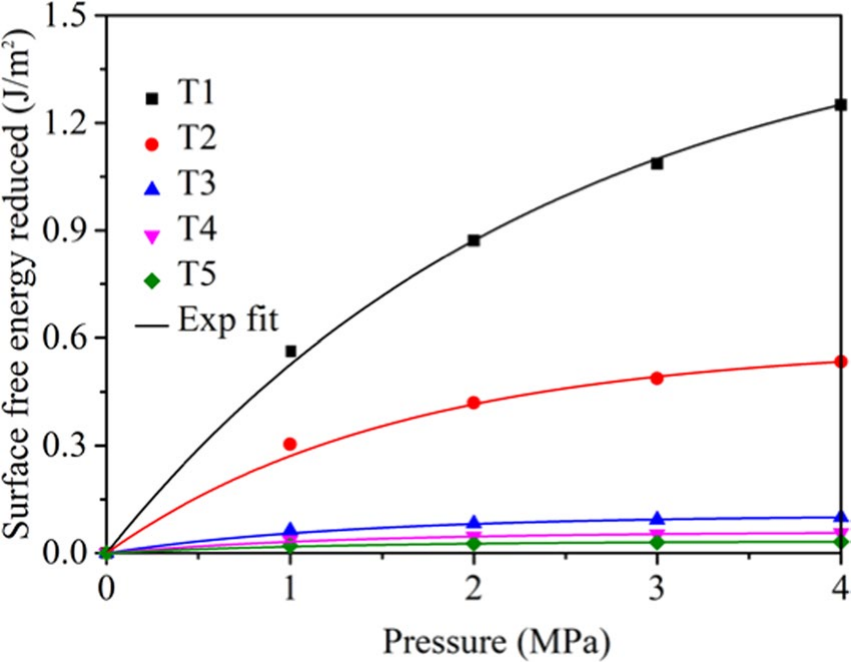
Reduction of surface free energy with pressure after electrochemical modification using four electrodes.

Formula (5) shows that the surface tension of coal depends on the adsorption capacity of CH_4_, the surface structure of coal, the temperature of coal-CH_4_ adsorption system and the pressure of CH_4_ gas. The reduction of surface free energy of coal can be calculated by formula (5). Figure 3 showed the reduction of surface free energy with pressure after electrochemical modification using four electrodes. It indicated that the reduction of surface free energy increased with increasing pressure, and after electrochemical modification using four electrodes, the reduction of surface free energy decreased, the reduction trend is T1 > T2 > T3 > T4 > T5. We can also observed that T3 and T4 decreased obviously compared with T2, this is because the surface free energy also related to specific surface area of coal as shown in Eq. (5). The specific surface area of T3 and T4 coal samples increased after electrochemical modification, leading to an obviously decrease of surface free energy.

## Discussion

When the non-inert metal electrodes, like aluminum, iron and copper, were used, the corrosion of anode occurs due to the following reactions:

6 $$M\to {M}^{n+}+n{e}^{-}$$

When a graphite electrode is applied, there is no corrosion reaction of graphite, but water is oxidized at anode.

7 $$2{H}_{2}O\to {O}_{2}(g)+4{H}^{+}+4{e}^{-}$$

The reactions occur at the cathode are the reduction reaction of metal or water.

8 $${M}^{n+}+n{e}^{-}\to M$$

9 $$2{H}_{2}O+2{e}^{-}\to 2O{H}^{-}+{H}_{2}(g)$$

**Figure 4:**
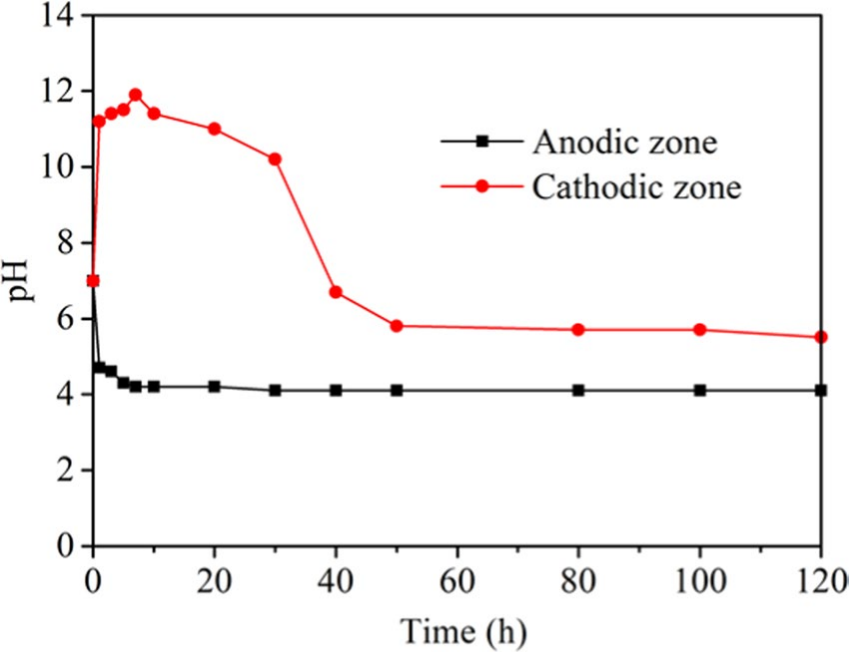
Dynamics of pH in anode and cathode areas during the electrochemical modification process.

The corrosion of anode produces M^n+^ ions, which move toward the cathode through ions migration. If M^n+^ ions presented at the cathode, the (8) reaction occurs. Theoretically, the decomposition potential of water is 1.23 V, and the actual decomposition potential is about 1.7 V^23^. In the electrochemical modification, the potential of the applied DC supply is 4.4 V, the reactions (6) and (8) will occur simultaneously with (7) and (9). The products of the electrode reactions move toward the counter electrodes, causing ion substitution in coal and the mineral matter. For example, H^+^ ions generated by the anode move toward the cathode, and the OH^−^ ions generated by the cathode move toward the anode. The mobility of H^+^ ions in the electrolyte is higher than that of OH^−^ ions. Since the coal and the mineral matter are negatively charged, the movement of H^+^ ions to the cathode is much higher than that of the electro osmotic flow. After electrochemical modification, the whole coal sample is more likely to be acidic, as shown in Fig. 4. This is due to coal particles generate less charge at lower pH value. Therefore, under higher pH conditions, OH^−^ is easier adsorbed on coal particles and increased the negative surface charge. As a result, solution monitoring shows that the cathode area is also acidic. The acidification modification increased the number of acidic groups on the surface of the coal samples, and significantly inhibits the capacity of gas adsorption while changing the surface area and pore volume^24^. The hydrogen over potential for graphite is greater than for metal electrodes, leading to hydrogen gas generation at graphite cathodes and accompanying higher H_3_O^+^ concentration, thus, the coal samples modified by graphite cathode is more acidic than that modified by metal electrodes. Chemical treatment like acid treatment to generate acidic surface also result in the decrease of methane adsorption^24,25^. Feng *et al*.^25^ use H_2_SO_4_, (NH_4_)_2_S_2_O_8_ and H_2_SO_4_/(NH_4_)_2_S_2_O_8_ acid to modified bituminous, respectively. After modification, the O-containing groups over the coal surface increased and methane adsorption decreased. The effect of acid modification of coal matrix on methane adsorption has been reported in literatures. H_2_SO_4_ and HNO_3_ acid treatment to generate acidic surface can reduce the CH_4_ adsorption in coal-based activated carbon was found by Zhang *et al*.^24^. Electrochemical modification is better than just chemical treatment because it not only increase acidic groups, also reaction with mineral matters in coal and migration them^6^.

**Figure 5:**
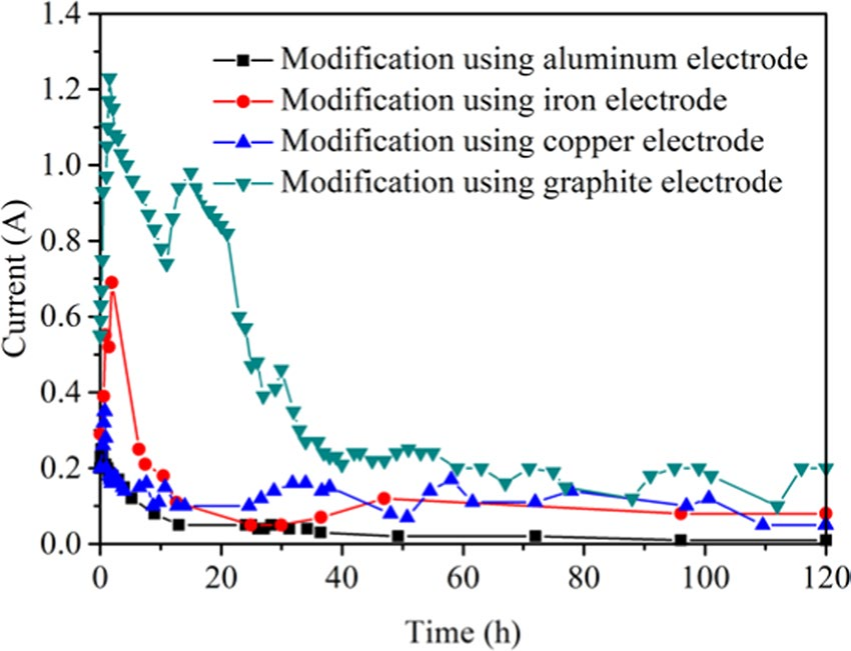
Dynamic of current during the modification process.

Figure 5 showed the dynamics electric current during the electrochemical modification using four electrodes, which indicated that the current increases first, then decreases, and finally tends to stable during the whole electrochemical modification process. The circuit current of electrochemical modification using graphite electrode is generally larger than that using non-inert metals electrodes, followed by copper and iron electrode, aluminum electrode least. The current-time curves were fitted by MATLAB and the magnitude of the charge transfer were calculated. The charge transfer of electrochemical modification using aluminum, iron, copper, and graphite electrodes electrode were 0.72 × 10^3^C/g, 2.28 × 10^3^C/g, 2.38 × 10^3^C/g, 2.80 × 10^3^C/g, respectively. The trend of calculated charge transfer is T2 < T3 < T4 < T5, which is in good agreement with the methane adsorption results. When graphite is used as the modified electrode, the current has three peaks, 1.23 A at 1.5 h, 0.98 A at 15.0 h and 0.46 A at 30.0 h, after that the current gradually decreased till 40.0 h, and finally stable at around 0.2 A. This is due to that after the DC applied, the electrochemical reaction occurs rapidly and the current increases, which leads to the first peak. The electrochemical reaction leads to the increase of temperature, when temperature increases, it causes a loss of electrical contact between soil and electrodes^26^. The contact loss leads to decrease of current. After the decrease of current, the electrochemical reaction weakens, and the temperature decreases, the contact increases again, and the current increases to a second peak. Because the graphite anode does not corrode, the O_2_ generated by the electrolytic water reaction diffuses into the atmosphere in the form of bubbles, which reduces the contact between the coal sample and the electrode plate and reduces the current. When the current decreases, the generated oxygen decreases, the contact between coal sample and electrode plate increases again, and the current increases, and the third peak appears.

**Figure 6:**
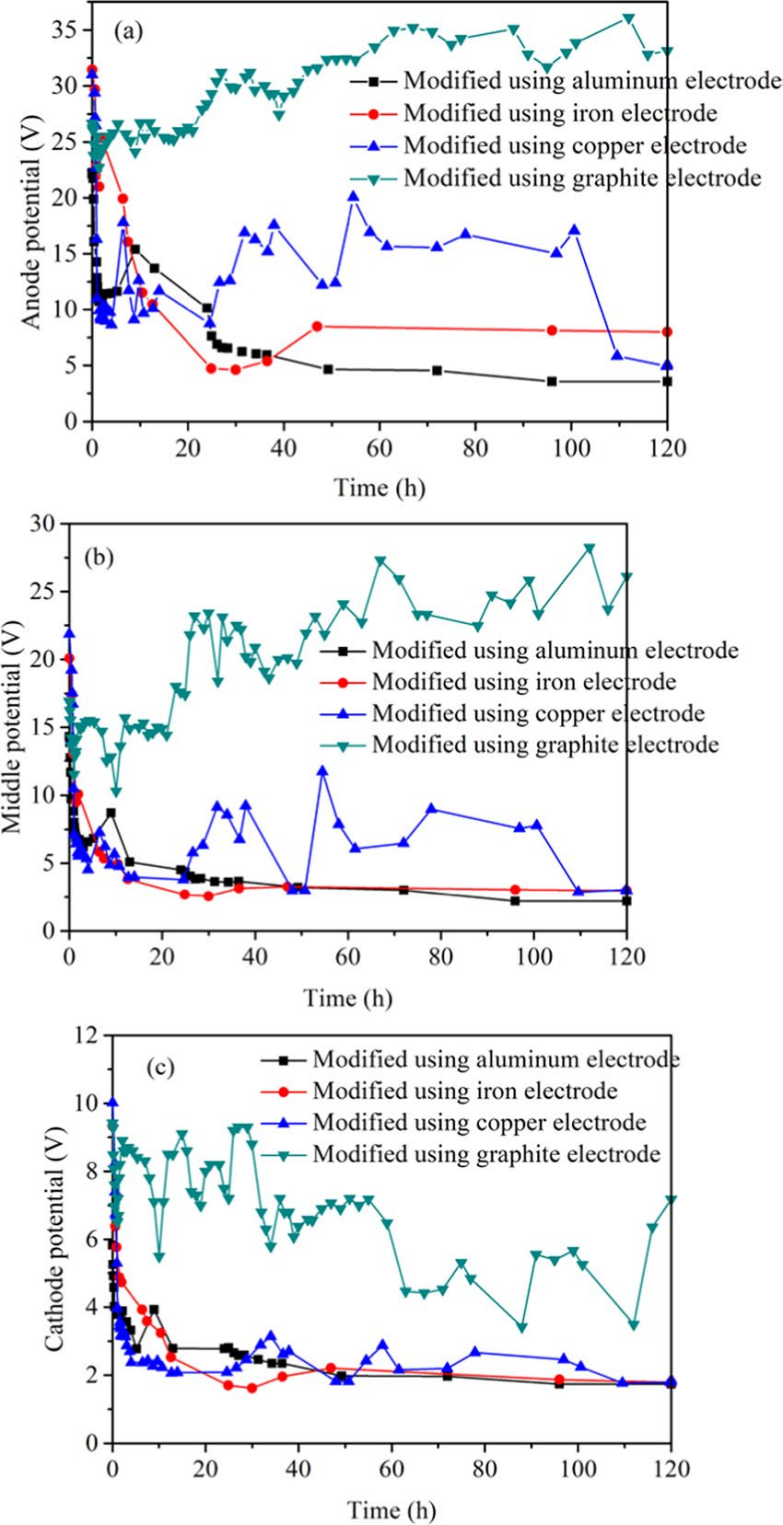
Change of potential with time during modification using different electrodes.

Figure 6 shows the variation of potential in the anode, middle and cathode regions with time during the modification of four kinds of electrodes. From (a), (b) and (c) of Fig. 6, it can be seen that in the whole process of electrochemical modification, the effective potential of graphite electrodes is the largest, followed by copper electrode and iron electrode, and aluminum electrodes least. The oxidation on the surface of copper, iron and aluminum electrodes and the separation of the plate from the cross section of the sample lead to a smaller potential than that of graphite electrodes. In addition, the increase of temperature would also result the loss of coal-electrode contact^26^. The electrochemical heating rises the temperature of coal sample and electrolyte, which leads to the rapid evaporation of water. The shrinkage of sample caused by the decrease of water content will inevitably lead to the reduction of contact between electrode plate and coal sample. In this experiment, only a small amount of decomposition of graphite electrode occurs, and the potential loss is basically stable. In the initial stage of modification, the copper electrode has a rapid decline, but then it tends to be stable. The reason is that the copper oxide formed on the surface of the copper electrode can also conduct electricity, which reduces the potential loss^14^. There is obvious oxidation on the surface of the electrode at the iron anode, which leads to the decrease of the potential. The passive film formed on the surface of the electrode at the aluminum anode hinders the effective transmission of potential, and the loss of potential is the greatest, which is basically consistent with the effect of electrochemical modification.

Moreover, when graphite was used as the modified electrode, the temperature of the modified system was substantially larger than the modification with copper, iron and aluminum electrode. The influence of temperature on electrochemical effect is mainly reflected in two aspects. On one hand, higher temperature increase the hydraulic conductivity and pore pressure, which results in improvement of migration of H^+^ and M^n+^ ions. For the other hand, higher temperature will further reduce methane adsorption in practical application of electrochemical treatment.

**Figure 7:**
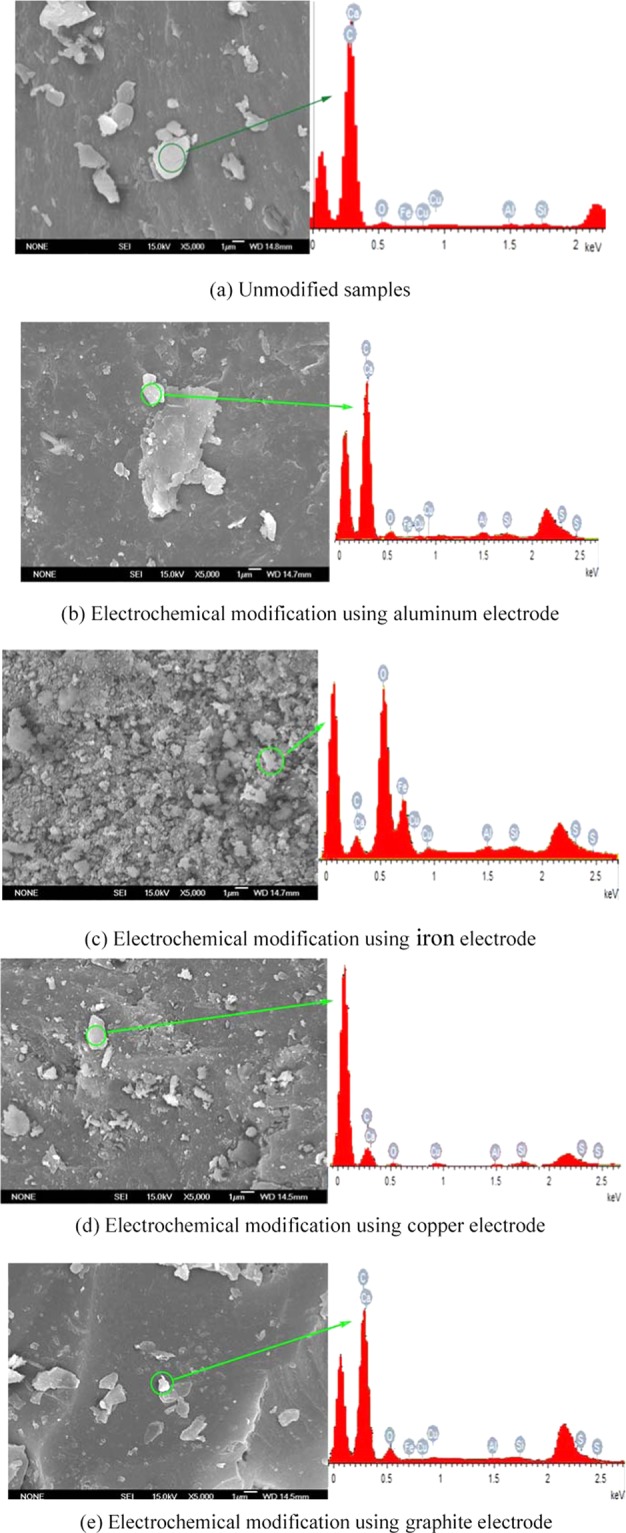
SEM and EDS images of anthracite unmodified and modified using different electrodes. (**a**) Unmodified samples. (**b**) Electrochemical modification using aluminum electrode. (**c**) Electrochemical modification using iron electrode. (**d**) Electrochemical modification using copper electrode. (**e**) Electrochemical modification using graphite electrode.

The methane adsorption in coal is mainly affected by the pore structures. The internal surface area of coal micro pores is directly related to the capacity of gas adsorption^27^. BET results shown in Table 4 indicated that the surface area of the unmodified coal sample was 6.5747 m^2^/g, the surface area increased to 6.5928 m^2^/g when the graphite electrode was used, and the impact is negligible. When the mental electrode was used, the surface area increased to 7.8845 m^2^/g, 22.1877 m^2^/g and 9.0359 m^2^/g modified with aluminum, iron and copper electrodes. For electrochemical modification using metal electrodes, the increase of surface area should be caused by metal nanoparticles. The increase of the surface area was due to the increase in the bond length of oxygen contents at the surface of the coal caused by the applied electric field^28^. The increase of surface area of coal samples will directly lead to the increase of gas adsorption. Although the surface area of the modified coal samples increased, the gas adsorption decreased after electrochemical treatment. The increased area was from the deposited metal species, like metal nanoparticles, as shown in Fig. 7(c). Moreover, coal samples were acidified by the H^+^ ions generated during the electrochemical modification, which inhibited the adsorption of gas. The inhibition of acidic groups on methane adsorption of coal samples is stronger than that of pore structures^24^.

**Table 4:** Pore structure changes after modification.

Test number (Electrode material)	Pore Size (nm)	Pore Volume (cm^3^/g)	Surface Area (m^2^/g)
T1(No electrode)	3.0402	0.004997	6.5747
T2(Aluminum)	3.3182	0.001429	7.8845
T3(Iron)	2.6397	0.005923	22.1877
T4(Copper)	3.7728	0.004711	9.0359
T5(Graphite)	2.8147	0.004639	6.5928

Figure 7 shown the SEM and EDS images of anthracite unmodified and modified using four electrodes. EDS can be used to analysis the mineral matters in coal-SEM-images^6^, and previous studies have shown that filling mineral matters in coal, such as kaolinite, telocollinite and clay minerals, can affect methane adsorption in coal^29,30^. Feng *et al*.^29^ pointed that the mesostructures of cell cavity pores with non-compact packing of the clay minerals appear to be the primary sites of methane adsorption in coal, and the telocollinite with fewer pores has a lower methane adsorption capacity. Therefore, EDS analysis can be used to distinguish mineral matters changes in coal after electrochemical modification, and further explain the effect of electrochemical modification on methane adsorption. After electrochemical modification using graphite electrode the clay minerals decreased most compared with metal electrodes, which further verification the methane adsorption decreased most using graphite electrode. According to the figure, the surface of the unmodified coal sample was adhered with minerals such as SiO_2_, Al_2_SO_3_ and FeS_2_. After electrochemical modification, the number and the size of mineral particles was decreased. For example, the FeS_2_ in the unmodified coal was 0.96%, which was reduced to 0.68% after modification with copper electrode, also decreased to 0.63% after modification with graphite electrode. The reason was that the H^+^ ion generated by the anode reactions made the electrolyte acidic, and the reactions of the acidic electrolyte and FeS_2_ was:

10 $${{\rm{FeS}}}_{2}+8{{\rm{H}}}_{2}{\rm{O}}\to {{\rm{Fe}}}^{3+}+2{{\rm{SO}}}_{4}^{2-}+16{{\rm{H}}}^{+}+15{{\rm{e}}}^{-}$$

The increase of Cu content in Fig. 5(b) can be explained by the intrusion of Cu^2+^ by the copper electrode reactions. Electrochemical modification using graphite electrode has almost the same surface area (only increased by 0.2%) and similar morphology as the unmodified sample, this is because that the increase of the surface area was due to the increase in the bond length of oxygen contents at the surface of the coal caused by the applied electric field, and the influences is limited. Moreover, effect of electrochemical modification on surface area of coal is relatively limited in fine coal samples, and it can only be reflected by SEM with lump anthracite due to the mineral-filled fractures of coal samples were unblocked by electrolytic reactions^6^. In our paper, before electrochemical modification, the coal samples were processed into fine particles of 0.18–0.25 mm according to GB/19560-2008. For fine coal samples, electrochemical modification has no obvious effect on the surface area (as shown by electrochemical modification using graphite electrode). For electrochemical modification using metal electrodes, the increase of surface area should be caused by metal nanoparticles, as shown in Fig. 7(c). Anthracite samples contain certain pyrite, which can be dissolved by H^+^ produced by electrolysis reaction and oxidized to Fe^3+^ under electrochemical actions. Under the action of electro migration, anthracite samples move to the cathode and combine with OH^−^ produced by electrolysis in the cathode area to form Fe(OH)_3_. When the iron electrode is used for electrochemically modification, the corrosion reaction of the iron anode will occur, resulting in a large number of Fe^3+^ ions intruding into the electrolyte, resulting in a large number of Fe(OH)_3_ flocculent precipitation attached to the surface of the coal sample, which is also the main reason for the larger test results of the iron electrode.

**Figure 8:**
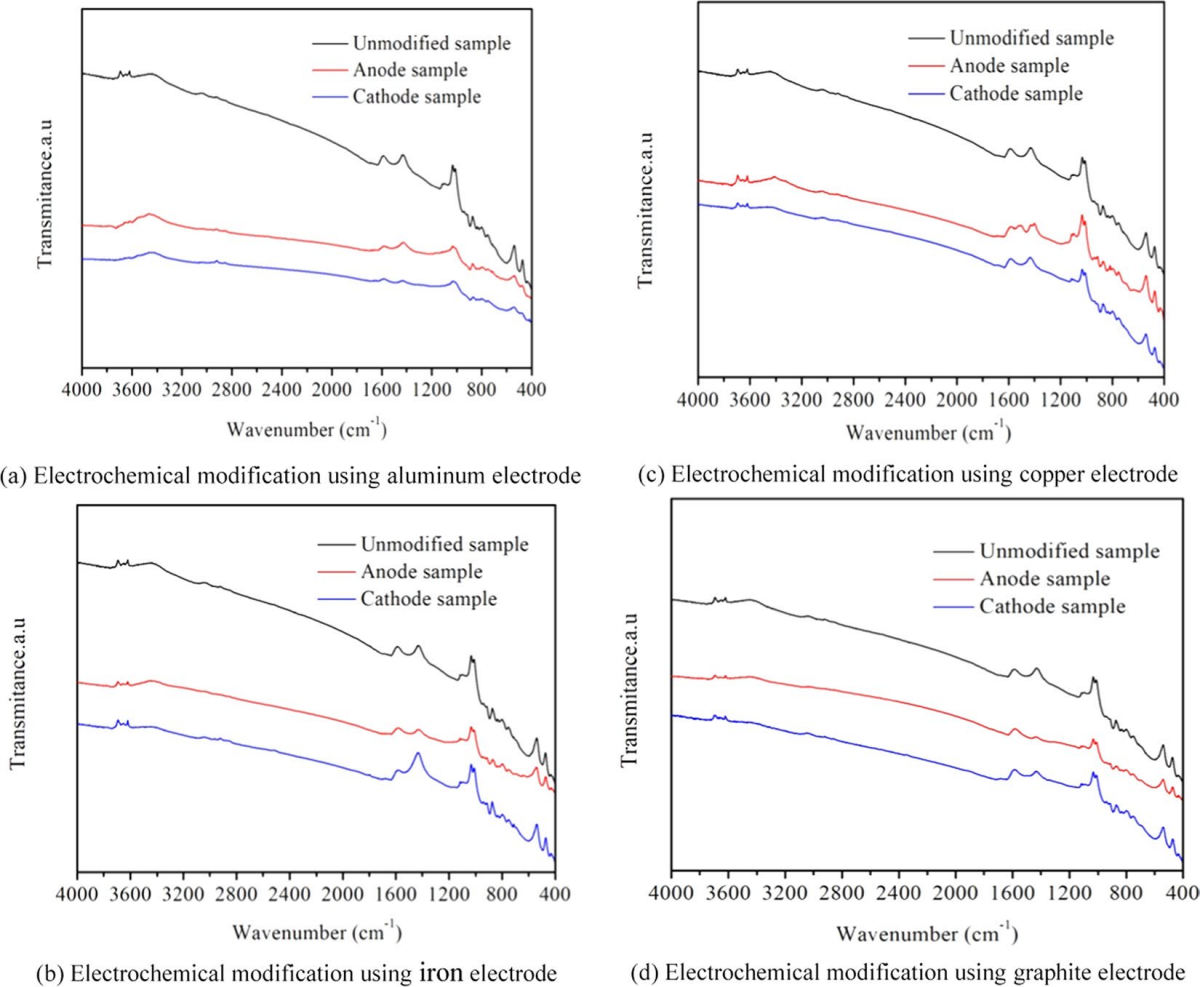
FTIR spectra of anthracite unmodified and modified using different electrodes. (**a**) Electrochemical modification using aluminum electrode. (**b**) Electrochemical modification using iron electrode. (**c**) Electrochemical modification using copper electrode. (**d**) Electrochemical modification using graphite electrode.

The surface characteristics of coal and the minerals in the coal also have significant influence on the adsorption capacity of gas^31–33^. Figure 8 showed the FTIR spectra of anthracite modified by different electrodes, it indicated that when copper electrode was used, the distinct changes of peaks mainly concentrated at 500–600 cm^−1^ and 1000–1200 cm^−1^, which are mainly derived as minerals in the coal^34,35^. After electrochemical modification, the peak at 1035 cm^−1^ decreased, and the peak at 1010 cm^−1^ increased, indicating that the Cu^2+^ produced by copper anode changed the mineral properties of coal. When graphite electrode was used, the peak at 3400 cm^−1^ of hydroxyl group decreased, the electrochemical actions are as follows:

11 $$RCO{O}^{-}+{R}^{1}CO{O}^{-}\to 2C{O}_{2}+R{R}^{1}+2{e}^{1}$$

The peak at 507 cm^−1^ and 1082 cm^−1^ of montmorillonite decreased due to the chemical interaction of H^+^ and montmorillonite. The peak at 876 cm^−1^ of calcite disappears was caused by H^+^ generated by anode reactions.

## Conclusions

1. Electrochemical modification can reduce the methane adsorption in anthracite effectively. The maximum adsorption capacity of unmodified anthracite was 39.920 ml/g, which decreased to 37.836 ml/g, 36.536 ml/g, 36.232 ml/g and 35.398 ml/g, after electrochemical modification using aluminum, iron, copper, graphite electrodes, respectively. The degree of influence is graphite > copper > iron > aluminum;
2. Electrodes have different effects on the potential well of methane adsorption of coal samples, which increased after electrochemical modification using the metal electrodes, and decreased using the graphite electrode.
3. The H^+^ generated during the process of electrochemical modification made the whole coal samples acidic, which increased the surface area and acidic groups of the coal samples. The increase in the surface area increased the adsorption of gas, however, the increase of the acidic groups reduced the gas adsorption, and the increase of acidic groups was dominant in the electrochemical modification;
4. The different electrode reactions were the main influence of the electrode materials on the electrochemical modification, the M^n+^ ions changes the properties of the clay mineral in the coal, and the H^+^ ions corrode the calcite minerals in the coal.

## Supplementary information

Supplementary Dataset 1
