# Supplementary material for: Experimental and mechanistic research on methane adsorption in anthracite modified by electrochemical treatment using selected electrode materials
Source: Sci Rep. 2019 Nov 20;9:17163. doi: 10.1038/s41598-019-53840-9 (PMC6868202; doi:10.1038/s41598-019-53840-9)
Supplement: Supplementary file 1 — Supplementary Dataset 1 [file 41598_2019_53840_MOESM1_ESM.pdf]

Experimental and mechanistic research on methane adsorption in anthracite modified by electrochemical treatment using selected electrode materials

Xiaoyu Zhang [1], Runxu Zhang [1], Tianhe Kang [1 *], Yaoqing Hu [1], Chao Li [1]

[1]Key Laboratory of In-situ Property-improving Mining of Ministry of Education, Taiyuan University of Technology, Taiyuan 030024, PR China

Xiaoyu Zhang, Email: tyutzhangxiaoyu@outlook.com

Runxu Zhang, Email: 18734902995@163.com

*Correspondence to Tianhe Kang, Email: kangtainhe@163.com

Yaoqing Hu, Email: yaoqinghu@yeah.net

Chao Li, Email: 18734132859@163.com

1. In view of the repeatability of methane adsorption results, we made repeated adsorption test for T1, T2, T3 and T4 to ensure the repeatability of the results as shown in Fig. 1(a), and we increased adsorption pressure to 10MPa as shown in Fig. 1(b). From comparison of the two times adsorption tests, we can ensure that the trend of T1>T2>T3>T4 is repeatable.

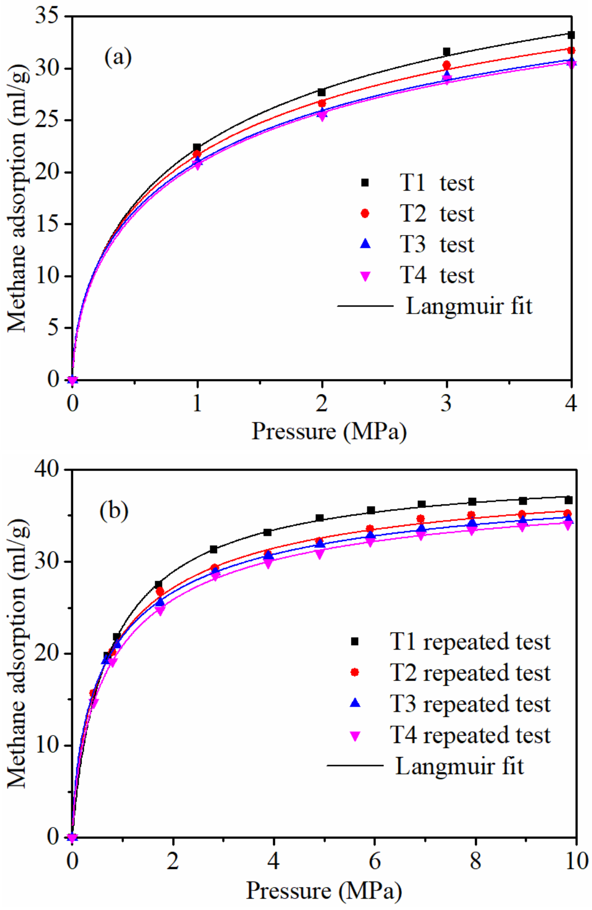

Figure. 1 Repeated testing of methane adsorption for T1, T2, T3 and T4.

2. In this experiment, Jincheng anthracite was used and the Zeta potential as a function of pH was tested as shown in Fig. 2. It indicated that the point of zero charge (PZC) is around pH 3.2. Coal particles generate less charge at lower pH than at higher pH. Therefore, under higher pH conditions, OH^- is easier to adsorb on coal particles and increase their negative surface charge. As a result, solution monitoring shows that the cathode area is also acidic.

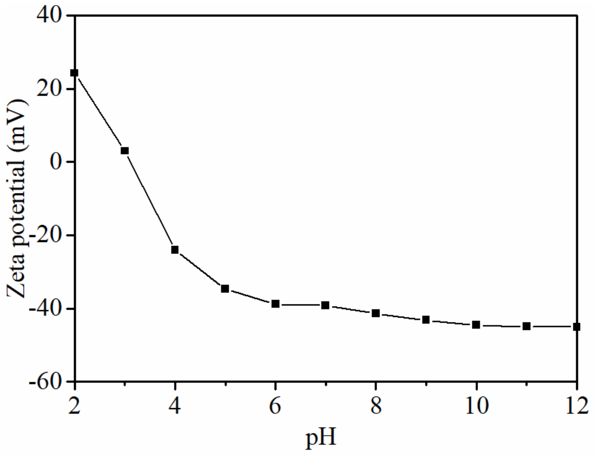

Figure. 2 Zeta potential of anthracite as a function of pH.

3. We have provided pore size distribution information of pore structure changes before and after electrochemical modification, as shown in Fig. 3.

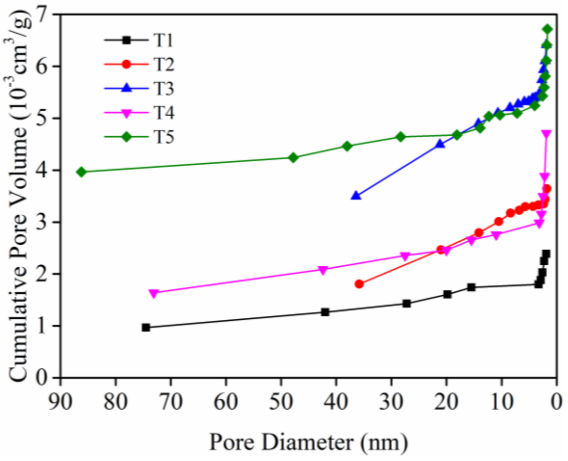

Figure. 3 Cumulative pore volume vs pore size after modification.

4. The reduction reaction occurs in cathode areas when metal electrodes were used. However, it cannot be reflected by EDS analysis, this is because only a few representative mineral points are selected for element analysis in the EDS test, which cannot reflect the reduction characteristics of the whole electrochemical process. For example, Fig. 4 (a, b) shows the representative SEM images of the anode and cathode electrochemical modification using iron electrode, respectively.

The EDS element weight content (%) is listed in Table 1. Among them, C is for $CaCO_3$, O is for SiO_2, Al is for Al_2O_3, Si is for SiO_2, S is for FeS_2, Ca is for Wollastonite, and some Fe and Cu. The content of Fe is higher at the anode, but not obvious at the cathode. Moreover, the reducibility can also by assistant analysis of the quality change of the electrode plates before and after electrochemical modification, and the quality of anode plate decreases and the quality of cathode plate increases, but the increase is very small and can be neglected. The reason may be that the migration ability of Fe^{3+} ions is poor, and the precipitation formed rapidly by oxidation of Fe^{3+} ions accumulates in the contact area between anode plate and coal sample, and cannot migrate quickly to the cathode.

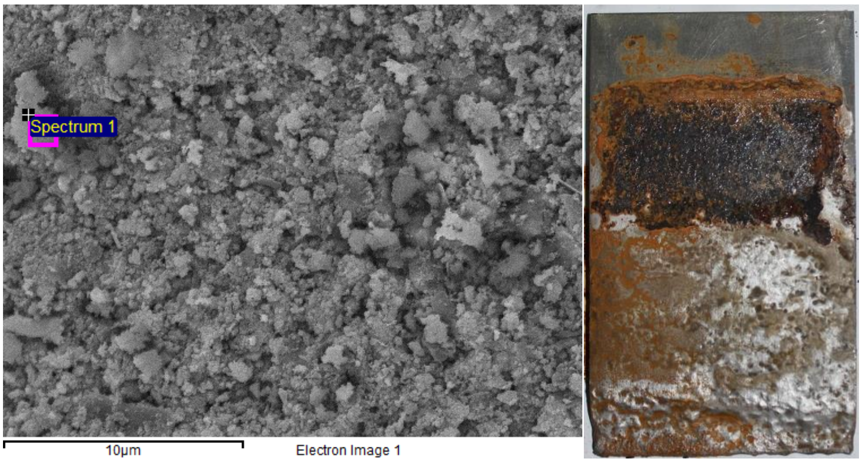

(a)

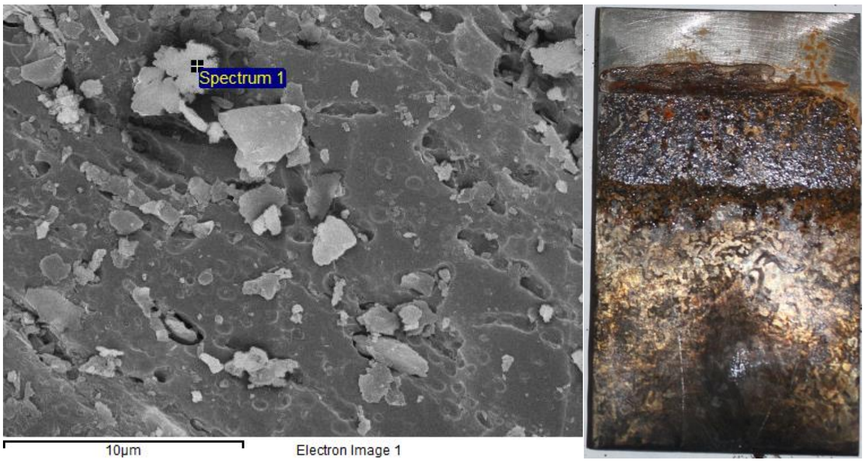

(b)

Figure. 4 SEM images and electrodes of the anode (a) and cathode (b) after electrochemical modification using iron electrode.

Table 1. The EDS element weight content (%)

Anode			Cathode		
Element	Weight%	Atomic%	Element	Weight%	Atomic%
C K	14.84	28.15	C K	47.65	67.36

O K	36.15	51.48	O K	23.53	24.98
Al K	0.62	0.52	Al K	0.32	0.20
Si K	0.86	0.70	Si K	0.51	0.31
S K	-0.69	-0.49	S K	-1.54	-0.82
Ca K	0.30	0.17	Ca K	0.38	0.16
Fe K	46.19	18.85	Fe K	0.30	0.09
Cu L	1.74	0.62	Cu L	28.85	7.71
Totals	100.00		Totals	100.00	
